# Exosomes Derived From Bone Mesenchymal Stem Cells Ameliorate Early Inflammatory Responses Following Traumatic Brain Injury

**DOI:** 10.3389/fnins.2019.00014

**Published:** 2019-01-24

**Authors:** Haoqi Ni, Su Yang, Felix Siaw-Debrah, Jiangnan Hu, Ke Wu, Zibin He, Jianjing Yang, Sishi Pan, Xiao Lin, Haotuo Ye, Zhu Xu, Fan Wang, Kunlin Jin, Qichuan Zhuge, Lijie Huang

**Affiliations:** ^1^Zhejiang Provincial Key Laboratory of Aging and Neurological Disorder Research, The First Affiliated Hospital of Wenzhou Medical University, Wenzhou, China; ^2^Department of Neurosurgery, The First Affiliated Hospital of Wenzhou Medical University, Wenzhou, China; ^3^Department of Pharmacology and Neuroscience, University of North Texas Health Science Center, Fort Worth, TX, United States

**Keywords:** traumatic brain injury, bone mesenchymal stem cells, exosomes, neuroprotection, microglia/macrophage, inflammation

## Abstract

Traumatic brain injury (TBI) is a leading cause of mortality and disability worldwide. Although treatment guidelines have been developed, no best treatment option or medicine for this condition exists. Recently, mesenchymal stem cells (MSCs)-derived exosomes have shown lots of promise for the treatment of brain disorders, with some results highlighting the neuroprotective effects through neurogenesis and angiogenesis after TBI. However, studies focusing on the role of exosomes in the early stages of neuroinflammation post-TBI are not sufficient. In this study, we investigated the role of bone mesenchymal stem cells (BMSCs)-exosomes in attenuating neuroinflammation at an early stage post-TBI and explored the potential regulatory neuroprotective mechanism. We administered 30 μg protein of BMSCs-exosomes or an equal volume of phosphate-buffered saline (PBS) via the retro-orbital route into C57BL/6 male mice 15 min after controlled cortical impact (CCI)-induced TBI. The results showed that the administration of BMSCs-exosomes reduced the lesion size and improved the neurobehavioral performance assessed by modified Neurological Severity Score (mNSS) and rotarod test. In addition, BMSCs-exosomes inhibited the expression of proapoptosis protein Bcl-2-associated X protein (BAX) and proinflammation cytokines, tumor necrosis factor-α (TNF-α) and interleukin (IL)-1β, while enhancing the expression of the anti-apoptosis protein B-cell lymphoma 2 (BCL-2). Furthermore, BMSCs-exosomes modulated microglia/macrophage polarization by downregulating the expression of inducible nitric oxide synthase (INOS) and upregulating the expression of clusters of differentiation 206 (CD206) and arginase-1 (Arg1). In summary, our result shows that BMSCs-exosomes serve a neuroprotective function by inhibiting early neuroinflammation in TBI mice through modulating the polarization of microglia/macrophages. Further research into this may serve as a potential therapeutic strategy for the future treatment of TBI.

## Introduction

Traumatic brain injury (TBI) is a leading cause of mortality and disability worldwide. Although treatment guidelines exist, the therapy of TBI remains a challenge for scientists and clinicians. To date, more than 30 clinical trials on promising drugs have failed to progress past phase II or phase III, making it impossible to single out the best treatment option for this condition (Xiong et al., 2013; Yang et al., 2017). Post-TBI MSCs therapy has received enormous attention in recent times, with various animal studies reporting neurological functional recovery after treatment and even with some studies already translated in the clinical setting (Zhang et al., 2008; Zhang H. et al., 2017; Cox et al., 2011). However, as with most novel treatments, there exist some limitations in their application in the clinical setting. A major setback of MSC therapy is the reported tendency of possible tumorigenicity (Jeong et al., 2011), with another being that only a small portion of transplanted MSCs survive and differentiate into neurons (Chopp and Li, 2002). In as much as MSCs are considered to have brain tissue repair properties, the underlying mechanism for their action remains unclear. Recently, some studies have discovered that the major mechanism of MSC-related tissue repair and function recovery was more likely due to their exosome-induced paracrine functions, not as a result of cell replacement (Li and Chopp, 2009; Lai et al., 2011).

Exosomes are lipid bilayer membrane vesicles, with a diameter of approximately 150 nm or less and are generated by all kinds of cells including MSCs, cancer cells and others (Costa-Silva et al., 2015; Zhang et al., 2015; Wu et al., 2018). Exosomes are rich in endosome-derived components such as mRNAs, microRNAs and proteins and are mostly characterized by transmission electron microscopy (TEM), marker proteins, and nanoparticle size analysis (Mincheva-Nilsson et al., 2006; Vlassov et al., 2012; Lotvall et al., 2014; Tkach and Thery, 2016). Currently, MSCs-exosomes have shown great promise as demonstrated by various findings in cell free therapy for cardiovascular disease, acute kidney injury, liver injury, lung injury, cutaneous wound healing, etc. (Rani et al., 2015). Compared to their parent cells, MSCs-exosomes have no proliferation and less immunogenicity due to little to no proliferative abilities and can be safely stored and delivered without losing functions (Lai et al., 2011).

Recent studies have mentioned the major contribution of MSCs-exosomes in improving neurobehavior performance, promoting neurogenesis and angiogenesis, and reducing inflammation after TBI (Zhang et al., 2015; Zhang Y. et al., 2017; Kim et al., 2016; Li et al., 2017). However, the mechanism underlying the anti-inflammation effects of exosomes remains unclear, with little evidence explaining the inflammatory changes at the early stage of TBI *in vivo*.

Inflammation is known to play a crucial role in the pathogenesis of TBI by aggravating the extent of brain injury via secondary injury (Woodcock and Morganti-Kossmann, 2013; Lozano et al., 2015; Chiu et al., 2016). After the TBI episode, direct impact of the trauma causes primary injury to the brain. Insight into TBI pathogenesis has identified the primary injury to trigger secondary injury cascades, which is characterized by excitotoxicity, oxidative stress, mitochondrial dysfunction, blood–brain barrier (BBB) disruption, and neuroinflammation. The initial inflammatory response is initiated to defend the injury site from invading pathogens and tissue debris. However, excess activation of neuroinflammation that involves microglia, astrocyte, other invading immune cells, cytokines, chemokines, and other inflammatory mediators accounts for majority of secondary cell death after TBI. Although microglia have been implicated in secondary injury, several studies have identified the transitioning of microglia during inflammation to present some benefit. For example, a transition of the microglia phenotype from pro-inflammatory M1 phenotype to anti-inflammatory M2 phenotype has been identified in some studies. This polarization effect has been seen to improve recovery after TBI, leading to the suggestion that it could be a possible treatment for TBI. In our present research, we found that exosomes derived from BMSCs could regulate the activation of different phenotypes microglia/macrophage cells in mice after TBI, which could be a potential mechanism of the functional recovery and neuroinflammation and apoptosis inhibition in the early stage induced by BMSC-exosomes.

## Materials and Methods

### Bone Mesenchymal Stem Cells Culture

BMSCs (P5) isolated from the Sprague-Dawley (SD) rat were purchased from Cyagen (Cyagen, RASMX-01101). Cyagen offered the identification results as follows: positive for CD29, CD44, and CD90 (>70%) and negative for CD34, CD45 and CD11b (<5%) in flow cytometry assays. BMSCs were cultured with DMEM medium (Gibco, C11995500BT) containing 10% fetal bovine serum (FBS; Gibco, 10099141, Austria) and 1% penicillin/streptomycin (HyClone, SV30010) in 75 cm^2^ culture flasks (Corning, 430641).

### Isolation and Identification of Exosomes

The isolation and purification of exosomes was performed as previously described (Mincheva-Nilsson et al., 2006). When cells reached 70–80% confluency, the culture medium was replaced with FBS-free medium, and BMSCs were cultured for an additional 48 h. Then, the media were collected, and exosomes were isolated by multistep centrifuging. Briefly, BMSCs culture FBS-free medium was collected and centrifuged at 300 *g* for 10 min and then for an additional 10 min at 2000 *g* to remove dead cells. Cells debris was removed by centrifuge at 10,000 *g* for 30 min. Then, the supernatants were ultracentrifuged at 110,000 *g* for 70 min at 4°C. The pellets were dissolved in PBS and ultracentrifuged at 110,000 *g* for another 70 min at 4°C. Finally, the final pellets isolated from each ten 75 cm^2^ culture flask were resuspended in 100 μl PBS.

The characteristics of the isolated exosomes were evaluated by western blot through the detection of CD63 (Mouse, Santa Cruz, sc-5275, 1:500), TSG101 (Rabbit, Abcam, ab125011, 1:1000) and Cytochrome c (Rabbit, CST, 11940s, 1:1000) expression (Lotvall et al., 2014). The morphology of the BMSCs-exosomes was assessed by TEM as previously described (Mincheva-Nilsson et al., 2006). The particle size distribution was analyzed by Zetasizer (Malvern, ZETASIZER Nano series-Nano-ZS) according to the manufacturer’s instructions.

### Animal Model and Treatments

Twelve- to fourteen-week-old male C57BL/6 mice were purchased from the Shanghai Laboratory Animal Center (Shanghai, China) and maintained in specific pathogen-free conditions in the Animal Center of Wenzhou Medical University. Animal welfare and experimental procedures were carried out in accordance with the Guide for the Care and Use of Animals (Animal Use and Care Committee of Wenzhou Medical University). The TBI model was induced by CCI as previously described (Romine et al., 2014; Perez et al., 2017). Briefly, mice were anesthetized with an intraperitoneal injection of 4% chloral hydrate (10 μl/g) diluted in normal saline (NS). With the mice fixed on a stereotactic frame (KOPF, Tujunga, CA, United States), the bregma was exposed and a 4 mm circular hole drilled around the center of lambda and bregma, which was 0.5 mm away from the midline. The mice were exposed to CCI injury at a velocity of 4 m/s, 1.0 mm depth, and 150 ms duration in the right hemisphere using Impact One^TM^ Stereotaxic Impactor for CCI (Leica, United States) with a 3 mm diameter piston (Romine et al., 2014). Sham animals underwent the same surgical procedure including the anesthesia and similar craniotomy without TBI.

C57BL/6 male mice (*n* = 59) were randomly divided into three groups: the Sham + PBS group (*n* = 17), TBI + phosphate-buffered saline (PBS) group (*n* = 21), and TBI + Exosomes group (*n* = 21). A total of 30 μg total protein of BMSCs-exosomes suspended in 150 μl PBS or equal volume PBS was administered by retro-orbital injection (Yardeni et al., 2011) 15 min after TBI. We performed the retro-orbital injection with an insulin syringe (Becton Dickinson, 328421) at an angle of approximately 30° into the medial canthus until the needle tip is at the base of the eye.

### Neurobehavioral Tests

The mNSS including sensory, motor, reflex, and balance tests were used to assess neurobehavior (Zhang et al., 2011; Wen et al., 2017). Neurological function was graded on a scale of 0–18 (normal score, 0; maximal deficit score, 18). Mice had been trained and assessed prior to surgery to ensure the normal score was 0. Then, neurobehavioral deficit scores were recorded by blinded tests at 1, 3, 7, and 14 days after TBI.

The rotarod test was carried out as described previously (Wang et al., 2015). In brief, mice were placed on a rotating drum with speed accelerating from 4 to 40 rpm within 5 min, and the time of the animal falling off the drum was recorded. The training began 3 days before TBI, and the mice underwent three trials every day. On the day of TBI, the animals performed three trials and the mean value was calculated as the baseline value for each animal. Then, the rotarod test was performed at 1, 3, 7, and 14 days post-TBI by a reviewer blinded to the animal groups.

### Lesion Identification by Hematoxylin-Eosin (H&E) Staining

Animals were anesthetized and then perfused transcardially with cold 0.9% saline followed by 4% paraformaldehyde (PFA) in PBS for fixation. The brains were harvested and postfixed in 4% PFA for 24 h and then dehydrated with 30% sucrose for 48 h. Dehydrated brains were embedded with optimal cutting temperature compound (OCT) for cutting. Serial coronal sections (10 μm thick) were cut using a cryostat (Leica Biosystems, CM1950) and stored at - 20°C. The section in the center of the injury has the lowest spared tissue and the largest lesion area. H&E staining was performed in the injury center using an H&E staining kit (Solarbio, China) according to the manufacturer’s instruction. The quantitation was done by NIH ImageJ.

### Western Blot

Proteins from the injured cortex were extracted in prechilled radio-immune precipitation assay buffer (RIPA) with phenylmethylsulfonyl fluoride (PMSF; Beyotime). The concentrations of proteins were measured with Pierce^TM^ BCA Protein Assay Kit (Thermo Scientific). Fifty micrograms of total proteins were separated using 12% (SDS-PAGE) and then transferred to PVDF membranes (Millipore Corp). The membranes were blocked with 5% milk for 2–3 h at room temperature and then incubated overnight with the following specific primary antibodies: β-actin (Rabbit, Affinity, AF7018, 1: 1000), BAX (Rabbit, Abcam, ab32503; 1:1000), BCL-2 (Rabbit, Abcam, ab182858; 1:1000). After washing with TBST, the bands were incubated with secondary antibodies for 1 h. Finally, the protein bands were visualized with ECL reagent.

### Real-Time Quantitative Polymerase Chain Reaction (PCR)

The injured cortex was separated from the mouse brain, and the total mRNA was isolated with Trizol (Thermo Fisher, 15596018). RevertAid First Strand cDNA Synthesis Kit (Thermo Fisher, K1622) was used to reverse transcribe 2 μg of total mRNA to cDNA. Then, SYBR Premix Ex Taq II (Tli RNase H Plus) (TaKaRa, RR820A) was used to perform real-time PCR. The controls of samples were normalized to GAPDH. The relative expression of different genes was calculated with ΔΔCt analysis. The primers used in real-time PCR were synthesized by Invitrogen (Shanghai, China) (Table 1).

**Table 1:** Sequences of primers used in real-time PCR.

Gene	Primer (5′-3′)
**TNF-α**	
Forward	CTGAACTTCGGGGTGATCGG
Reverse	GGCTTGTCACTCGAATTTTGAGA
**IL-1β**	
Forward	ATTGTGGCTGTGGAGAAG
Reverse	AAGATGAAGGAAAAGAAGGTG
**Arg-1**	
Forward	AACACGGCAGTGGCTTTAACC
Reverse	GGTTTTCATGTGGCGCATTC
**INOS**	
Forward	ATGTCCGAAGCAAACATCAC
Reverse	TAATGTCCAGGAAGTAGGTG
**GAPDH**	
Forward	AAGAAGGTGGTGAAGCAGG
Reverse	GAAGGTGGAAGAGTGGGAGT


### Immunofluorescence

To assess the activation and polarization of microglia/ macrophage, brain sections were processed for immunostaining with antibody against iba1 (Goat, Abcam, ab5076, 1:800), iNOS (Rabbit, Abcam, ab15323, 1:300), CD206 (Mouse, Abcam, ab8918, 1:300). Brain sections were washed with PBS three times and then incubated in the blocking solution (0.4% Triton X-100, and 10% donkey serum in PBS) for 1 h at room temperature, followed by incubation with primary antibody at 4°C overnight and another 30 min at 37°C. Sections were then washed with PBS three times and incubated with the secondary antibody at 37°C for 1 h. Sections were then washed with PBS and treated with DAPI for 5 min and then sections mounted by Antifade Mounting Medium (Beyotime, P0126) for preserving the fluorescence (Yang et al., 2016). Images were taken using a scanning-fluorescence microscope (Leica Microsystems). Analyses from three randomly selected stained slices of brain tissue per mouse was performed. Four digital microscopic images around the marginal zone of the lesion area in cortex of each slice were randomly applied. Double positive cells were counted, and the average cell number of each group was calculated.

### Statistical Analysis

Statistical testing was performed using IBM SPSS Statistics version 21.0. Data were presented as the mean ± standard error of the mean (SEM). Differences between two groups were examined with an unpaired Student’s *t*-test. One-way ANOVA was used to compare multiple groups. LSD *post hoc* analysis was used to analyze results from the rotarod test and expression of Bax, Bcl2, INOS and Arg-1 levels, and Dunnett T3 *post hoc* analysis was used in mNSS assessment and IL-1β and TNF-α expression study. One-way repeated measures ANOVA was also performed to analyze neurobehavior tests. The results were considered significant when *P*-value < 0.05.

## Results

### Characterization of BMSC-Derived Exosomes

**FIGURE 1:**
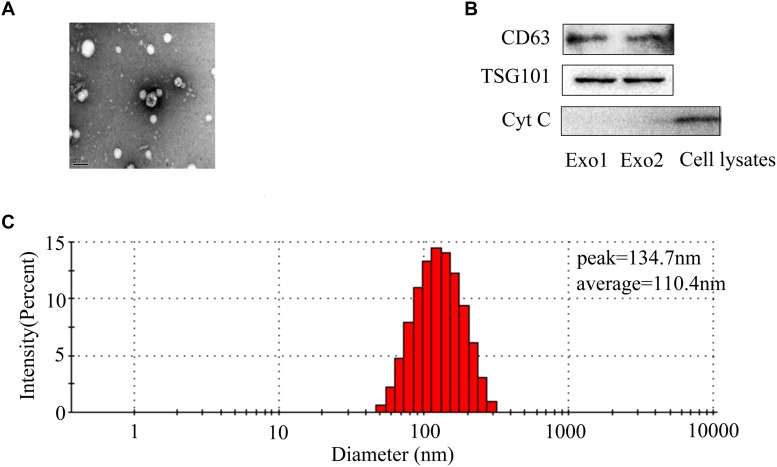
Identification of BMSCs-exosomes. **(A)** Transmission electron microscopy image of BMSCs-exosomes. Scale bar: 100 nm. **(B)** Exosomal markers (CD63, TSG101) and cytochrome c were analyzed by western blot. **(C)** Nanoparticles detection was performed to analyze particle size of exosomes. The percentage population of BMSC-exosomes by counts was shown.

To better understand the characterization of exosomes, exosomes were assessed by TEM and western blot. TEM revealed the existence of exosomes, small membrane vesicles sized from 30 to 150 nm (Figure 1A). The isolated BMSCs-exosomes were found to express high levels of TSG101 and CD63. There was almost no expression of Cytochrome c as a negative control (Figure 1B). The particle size detection showed that BMSCs generate particles with a peak diameter at 134.7 nm and an average diameter of 110.4 nm (Figure 1C).

### BMSC-Derived Exosomes Improved Functional Recovery After TBI

**FIGURE 2:**
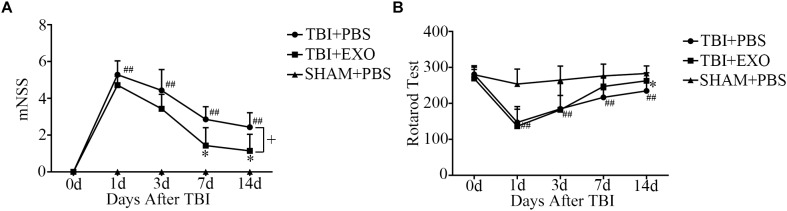
BMSCs-exosomes improve function recovery post-TBI. Twelve to fourteen-week-old male C57BL/6 mice were subjected to TBI by CCI and treated by BMSCs-exosomes through retro-orbital injection. The neurobehavior was evaluated from day 1 to day 14 post-TBI by mNSS and Rotarod Test **(A,B)**. ^∗^*P* < 0.05 versus the TBI + PBS group, ^##^*P* < 0.01 versus the SHAM + PBS group; one-way ANOVA. ^+^*P* < 0.05; one-way repeated measures ANOVA with Dunnett T3 *post hoc* test for **(A,B)** (*n* = 7).

After TBI, the mice demonstrated obvious behavioral deficiencies. Although some degree of spontaneous functional recovery was observed, we found that BMSCs-exosome treatment after TBI could accelerate function recovery. As shown by the mNSS test, treatment with exosomes resulted in a significant improvement of neurological function compared with the control group after TBI at 7 and 14 days (Figure 2A). Treatment with BMSCs-exosomes improved the performance of the TBI mice on the rotarod test at 14 days post-TBI (*P* < 0.05; *n* = 7) compared with the TBI mice treated with PBS (Figure 2B).

### BMSC-Derived Exosomes Reduced Lesion Area After TBI

**FIGURE 3:**
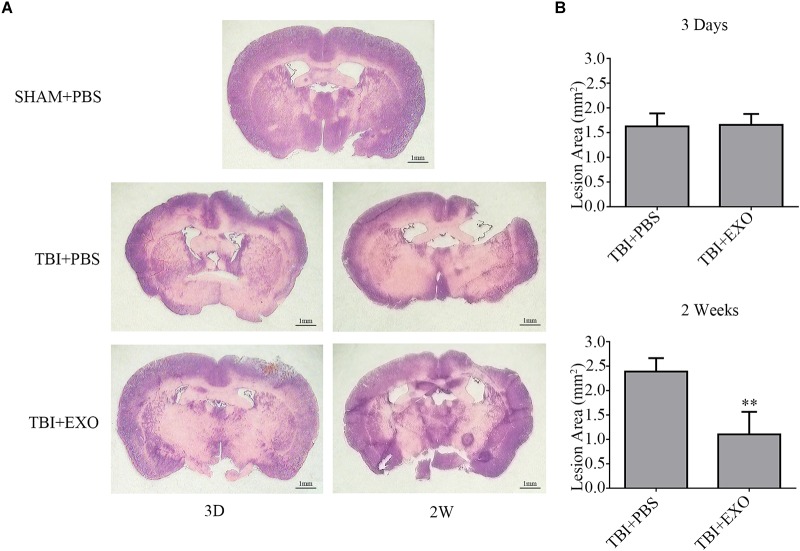
BMSCs-exosomes reduce cortical lesion post-TBI. **(A)** H&E staining of brain tissue from the TBI + PBS group at 14 days post-TBI showed serious tissue lesion, whereas reduced tissue lesion was observed in the TBI + EXO group. **(B)** The mean (±SEM) of cortical lesion area (mm^2^) after controlled cortical impact injury. ^∗∗^*P* < 0.01 versus the TBI + PBS group (*n* = 4).

To further illuminate the beneficial effects of exosome treatment, H&E staining of brain tissue section was performed at 3 and 14 days after TBI, with mice sacrificed 3 days after scalp incision as the control group. As is shown, the brain tissue loss of the exosome treatment group was much less than that in the PBS treatment group at 14 days after TBI (Figure 3A). To be sure of the variation between the two groups, quantification of the lesion area was performed. Lesions at 14 days post-TBI were much bigger than those 3 days post-TBI in the PBS treatment group (*n* = 4, *P* < 0.01). However, exosome treatment at 14 days post-TBI significantly reduced the lesion area compared with PBS treatment (*P* < 0.01) and appeared better than 3 days post-TBI by unpaired Student’s *t*-test (*P* = 0.074; Figure 3B).

### BMSC-Derived Exosomes Attenuated Cell Apoptosis and Inflammation After TBI

**FIGURE 4:**
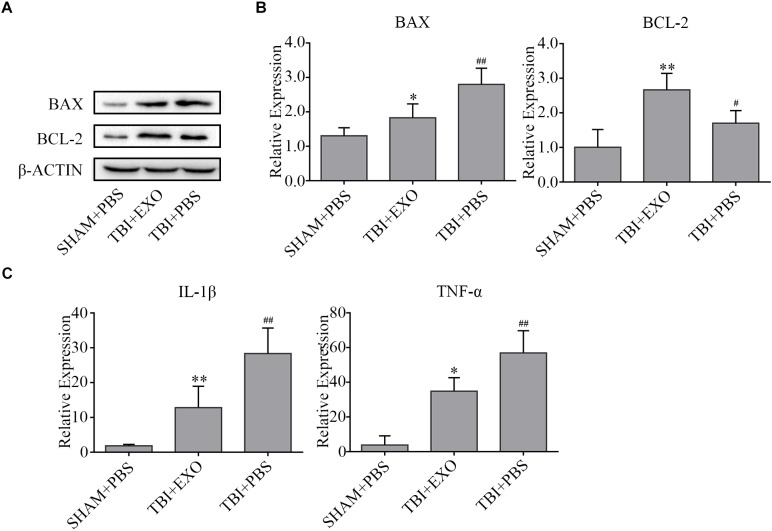
BMSCs-exosomes affect cell death and inflammation post-TBI. **(A)** The apoptosis associated proteins Bax and Bcl-2 were analyzed by western blot at 3 days after TBI. β-actin was used as an internal control (*n* = 4). **(B)** The mRNA expressions of pro-inflammatory cytokines (IL-1β and TNF-α) were analyzed by real-time PCR and normalized with GAPDH mRNA levels at 1 day after TBI (*n* = 6). ^∗^*P* < 0.05, ^∗∗^*P* < 0.01 versus the TBI + PBS group, ^#^*P* < 0.05, ^##^*P* < 0.01 versus the SHAM + PBS group **(C)**.

Western blot results revealed that the expression of Bcl-2 was upregulated, whereas Bax was downregulated in the exosome treatment group compared to the PBS control group at 3 days after TBI, suggesting that BMSCs-exosomes attenuated cell apoptosis (Figure 4A). The RNA expression levels of proinflammation cytokines (IL-1β, TNF-α) were notably inhibited by exosome treatment compared to that of the PBS control group at 1 day after TBI, indicating that BMSCs-exosomes attenuated inflammation (Figure 4B).

### BMSC-Derived Exosomes Decreased the Activation of Microglia/Macrophage M1 Phenotype Cells but Increased M2 Phenotype After TBI

**FIGURE 5:**
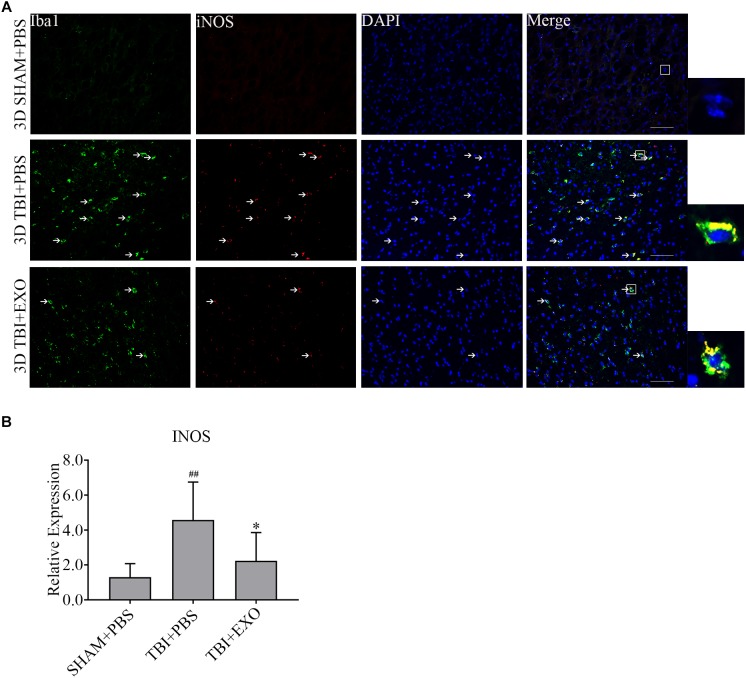
BMSCs-exosomes inhibit the M1 polarization of microglia/macrophage at 3 days post-TBI. **(A)** Compared with TBI + PBS group, INOS+/iba1+ cells had significantly decreased in the TBI + EXO group at 3 days after TBI. Arrows indicate iNOS+/Iba1+ cells. Scale bar = 50 μm (*n* = 4). **(B)** The mRNA expressions of INOS (*n* = 6). ^∗^*P* < 0.05 versus the TBI + PBS group, ^##^*P* < 0.01 versus the SHAM + PBS group.

**FIGURE 6:**
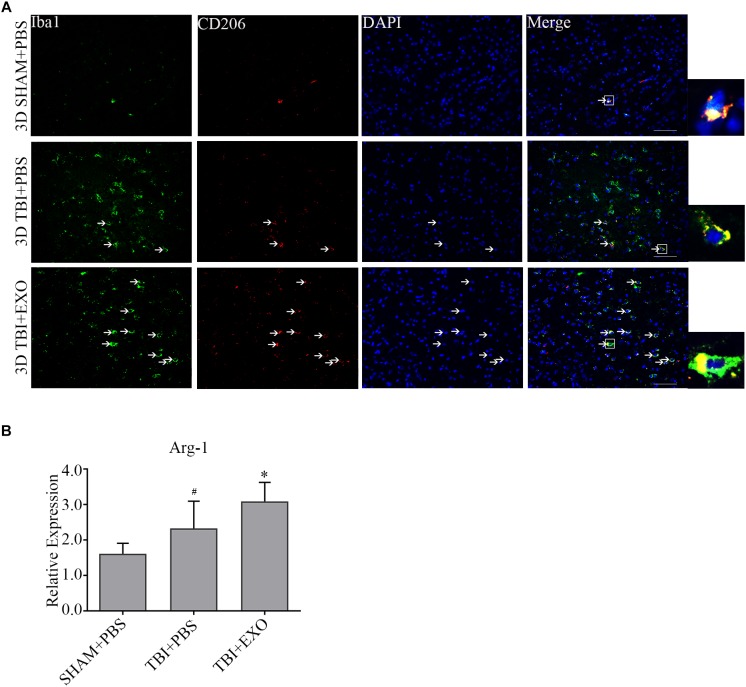
BMSCs-exosomes promote the M2 polarization of microglia/macrophage at 3 days post-TBI. **(A)** Compared with TBI + PBS group, CD206+/iba1+ cells had significantly increased in the TBI + EXO group at 3 days after TBI. Arrows indicate CD206+/Iba1+ cells. Scale bar = 50 μm (*n* = 4). **(B)** The mRNA expressions of arg-1 (*n* = 6). ^∗^*P* < 0.05 versus the TBI+PBS group, ^#^*P* < 0.05 versus the SHAM + PBS group.

To explore the possible mechanism of the recovery induced by BMSCs exosomes, we performed immunofluorescence and real-time PCR to detect the M1 polarization of microglia/macrophage cells. The results showed the M1 polarization marker iNOS was downregulated at 1 day after exosomes administration by real-time PCR (Figure 5B). The co-stained brain sections showed that the iNOS^+^/Iba1^+^ cells around the lesion area were decreased by exosome treatment at 3 days after TBI (Figure 5A). Meanwhile, the activation of M2 cells was also detected. The results of the real-time PCR showed that exosome treatment could significantly increase Arg-1 expression at 1 day after TBI (Figure 6B). At the tissue level, the increased CD206+/Iba1+ cells were detected by immunofluorescence in the TBI+EXO group compared to the TBI+PBS group (Figure 6A).

## Discussion

In this study, we demonstrated that the early systemic administration of BMSCs-exosomes showed promising therapeutic effects at early stages of TBI. The exosome treatment group had the following significant improvements compared to the PBS treatment group: (1) improved functional recovery; (2) reduced cortical lesion volume; (3) attenuated cellular apoptosis; and (4) modulated neuroinflammation, including the cytokines levels and polarization of microglia/macrophage.

We hold the view that the anti-inflammatory function of exosomes played a vital role in functional improvement even at 7 days in the exosomes treated group. As mentioned earlier, inflammation plays a crucial role in the pathogenesis of TBI. Both primary and secondary insults activate the release of cellular mediators including pro-inflammatory cytokines, prostaglandins, free radicals, and complement, which upregulate chemokines and adhesion molecules and in turn mobilize immune cells and glia cells. Noted among these mediators are leucocytes, macrophages and T-cell lymphocytes cells, which migrate from the peripheral immune system to the injury tissue, along with the activation of microglia, macrophage and other glia cells, promoting the inflammation cascade. Although early activation of inflammation presents some benefit of eliminating injured tissues and debris, excessive and continuous systemic inflammatory cascades induce diffuse axonal injury, contusion, intracranial hemorrhage and brain edema, which usually lead to immediate cell death. Furthermore, progressive neurodegeneration and delayed cell death might also result from excessive inflammation after TBI (Lucas et al., 2006; Werner and Engelhard, 2007; Lozano et al., 2015; Yang et al., 2017). Our results showed a greater loss of brain tissue at the injured area after 14 days compared to day 3 without any treatment, an expected result consistent with the secondary injury process after TBI. After a TBI episode, exacerbated secondary injury results in excessive neuronal cell death if there is no intervention. As such, more and more brain tissue is expected to be lost in and around injury site, as was seen in our findings. The administration of exosomes was seen to reduce brain tissue loss when compared with PBS group at 14 days, an observation that could be explained by the anti-inflammatory properties of exosomes in deferring or attenuating second injury and hence cell death and tissue loss.

To achieve a clearer picture of the mechanism leading to the function recovery, apoptotic and inflammatory marker levels were detected. Apoptosis is primarily regulated by the upstream Bcl-2 family and the downstream caspase family, therefore Bcl-2 (anti-apoptotic protein) and Bax (pro-apoptotic protein) are markers useful in detecting this programmed cell death (Cory and Adams, 2002). In our study, BMSCs-exosomes were seen to significantly increase the levels of Bcl2 while decreasing the amount of Bax expression (Figures 4A,B), similar to what was reported by Huang et al. (2017) in spinal cord injury.

Microglia/macrophages are activated during brain injury. Once activated, these cells are thought to be a major sources of both proinflammatory cytokines and chemokines in the CNS, including the release of TNF-α and IL-1β (John et al., 2003). The recruited macrophages and activated microglia cells mount a protective response against TBI. However, when microglia/macrophage activation become excessive after TBI, the cytokines and chemokines released by microglia/macrophages can directly or indirectly cause damage to neural cells, increase the permeability of the BBB, activate astrocytes and recruit leukocytes. This results lead to an amplification of the inflammation and cell death (Herx and Yong, 2001; Stoll et al., 2002; Chodobski et al., 2011; Hernandez-Ontiveros et al., 2013). TNF-α and IL-1β are important pro-inflammation cytokines, which are released within several hours after TBI. These cytokines are thought to be stored in an inactive state in the cell as precursor proteins but eventually get activated into active molecules after cell damage at the initial injury stage (Morganti-Kossmann et al., 2002; John et al., 2003; Liberto et al., 2004). We found that exosome treatment could inhibit the expression of TNF-α and IL-1β 1 day after TBI (Figure 4C), further consolidating the anti-inflammatory property of BMSCs-exosomes and the ability to promote cell survival.

Although microglia have been implicated in exaggerating the inflammatory response after brain injury, the current findings highlighted a key role of microglia in neuroprotection. For example, some studies have identified transitioning to occur between the two main subtypes of microglia, namely, M1 and M2. These studies reported polarization from the pro-inflammatory phenotype M1 to the anti-inflammatory phenotype M2, which suppresses M1 pro-inflammatory mediators leading to tissue repair (Miron et al., 2013; Chiu et al., 2016). Recent studies have shown that stem cells-generated exosomes could reduce the number of CD68+ microglia/macrophage cells, the M1 phenotype microglia/macrophage cells, after TBI (Zhang et al., 2015; Li et al., 2017). Li et al. (2017) also found odontogenic stem cell-exosomes could modulate microglia/macrophage polarization directly *in vitro*. In this study, our results showed that the administration of BMSCs-exosomes could promote the transition of the M1 phenotype microglia/macrophage to the M2 phenotype *in vivo* (Figures 5, 6), which was displayed by the reduction of the pro-inflammation cytokines (IL-1β, TNF-α) (Figure 4), indicating the interruption of the inflammation cascade process and tissue damage in an early stage.

Exosome treatment could significantly improve functional recovery and reduce the lesion volume after TBI, as seen in our studies. Similarly, Xin et al. (2013) found improvement in stroke outcome when exosomes generated from MSCs with green fluorescent Protein (GFP) were taken up by astrocytes and neurons after intravenous injection of MSCs 24 h post stroke in rats. Furthermore, they also demonstrated that systemic injection of 100 μg exosomes 24 h post-TBI could improve neurobehavior via angiogenesis and neurogenesis and neuroinflammation reduction (Zhang et al., 2015). Another study found that intravenous injection of 30 μg exosomes 1 h post-TBI could reduce inflammation and improve function recovery in mice (Kim et al., 2016). Having established the potential of exosomes in improving post-TBI recovery, there remain some unanswered questions. For example, although Xin et al.’s (2013) work demonstrated that exosomes released by MSCs could be captured by astrocytes and neurons, the level of exosomes reaching the brain and producing therapeutic effects remains unknown. According to Kim et al.’s (2016) work, the suppression of neuroinflammation by exosomes was dose dependent; there is therefore the need to ascertain whether doses greater than 30 μg protein have more significant therapeutic effects. Systemic injections of exosomes through veins resulted in promising function recovery in TBI animals, even in swine (Xin et al., 2013; Zhang et al., 2015; Kim et al., 2016; Zhang Y. et al., 2017; Williams et al., 2018). However, according to Matthew’s study, intravenous injections has approximately 1% or less exosomes accumulation in the brains of mice (Wiklander et al., 2015). However, further investigation is warranted to determine whether TBI would influence the biodistribution of exosomes, because of the broken BBB and activated immune cells. Intra-arterial or intracerebral injection is another possibility in terms of drug administration for better accumulation in the brain. A study by Walker et al. (2012) indicated that MSCs therapy after TBI might act as remote bioreactors stimulating lung macrophages and spleen T regulatory cells, increasing circulating anti-inflammatory cytokines. Most exosomes are also trapped in the lung and spleen (except liver) and might also have remote effects on the brain (Wiklander et al., 2015). Finally, the optimum time for exosomes administration post-TBI should also be investigated. Exosome administration 24 h post-TBI has proven to improve angiogenesis and neurogenesis and reduce neuroinflammation. Our study showed that the application of exosomes at a super-early stage 15 min post-TBI could at least interrupt the cycle of tissue destruction and inflammation at an early stage. However, it is unknown if the administration of exosomes at the early stage or the later stage could yield a more effective result. These are some of the questions that need to be answered to make the application of exosomes in the treatment of TBI more effective.

## Conclusion

In summary, our results indicate that BMSCs-exosomes exert a neuroprotective effect by inhibiting early neuroinflammation in mice with CCI-induced TBI via modulating microglia/macrophage polarization. Therefore, the use of BMSCs-exosomes might serve as a promising alternative therapy for the treatment of TBI by attenuating early neuroinflammation.

## Author Contributions

LH and QZ designed the experiments and edited the manuscript. KJ gave guidance to the experiments. HN performed the experiments, analyzed the data, and wrote the manuscript. SY and ZH interpreted the data and prepared the figures. FS-D, JH, and JY wrote the manuscript. KW, SP, XL, HY, ZX, and FW performed the experiments.

## Conflict of Interest Statement

The authors declare that the research was conducted in the absence of any commercial or financial relationships that could be construed as a potential conflict of interest.
